# What is different about living alone with cancer in older age? A qualitative study of experiences and preferences for care

**DOI:** 10.1186/1471-2296-14-22

**Published:** 2013-02-20

**Authors:** Barbara Hanratty, Julia Addington-Hall, Antony Arthur, Lucy Cooper, Gunn Grande, Sheila Payne, Jane Seymour

**Affiliations:** 1 Hull York Medical School, Department of Health Sciences, University of York, Seebholm Rowntree Building, Heslington, York YO10 5DD, UK; 2 Faculty of Health Sciences, University of Southampton, Southampton, UK; 3 School of Nursing Sciences, University of East Anglia, Norwich, UK; 4 Department of Public Health and Policy, University of Liverpool, Liverpool L69 3GB, UK; 5 School of Nursing, Midwifery and Social Work, University of Manchester, Manchester, UK; 6 Division of Health Research, Lancaster University, Lancaster, UK; 7 School of Nursing, Midwifery and Physiotherapy, University of Nottingham, Nottingham, UK

**Keywords:** Living arrangements, Aged, Aged, 80 and over, Health services for the aged, Neoplasms, Palliative care, Terminal care, Advanced care planning

## Abstract

**Background:**

Increasing numbers of older patients with advanced cancer live alone but there is little research on how well health services meet their needs. The aim of this study was to compare the experiences and future preferences for care between two groups of older people with cancer in their last year of life; those who live alone, and those who live with co-resident carers.

**Methods:**

In-depth qualitative interviews were conducted with 32 people aged between 70 and 95 years who were living with cancer. They were recruited from general practices and hospice day care, when the responsible health professional answered no to the question, of whether they would be surprised if the patient died within twelve months. Twenty participants lived alone. Interviews were recorded and transcribed and the data analysed using a Framework approach, focussing on the differences and commonalities between the two groups.

**Results:**

Many experiences were common to all participants, but had broader consequences for people who lived alone. Five themes are presented from the data: a perception that it is a disadvantage to live alone as a patient, the importance of relational continuity with health professionals, informal appraisal of care, place of care and future plans. People who lived alone perceived emotional and practical barriers to accessing care, and many shared an anxiety that they would have to move into a care home. Participants were concerned with remaining life, and all who lived alone had made plans for death but not for dying. Uncertainty of timescales and a desire to wait until they knew that death was imminent were some of the reasons given for not planning for future care needs.

**Conclusions:**

Older people who live alone with cancer have emotional and practical concerns that are overlooked by their professional carers. Discussion and planning for the future, along with continuity in primary care may hold the key to enhancing end-of-life care for this group of patients.

## Background

People with cancer are one of the largest sections of the older population with end-of-life care needs, and many are living alone. Around half of UK residents over the age of 65 years live in single person households [1]; three quarters of all cancer deaths occur in this age group [2] and more than 1.3 million people aged over 65 years have received a cancer diagnosis [3]. Living alone with cancer is known to be associated with worse quality of life, higher levels of distress [4,5], and a greater risk of not dying at home [6,7]. Living arrangements do influence end-of-life preferences [8], but much of the empirical work has focussed on place of death, which may have different influences, meaning and stability, compared to place of care [9-11]. Families are known to be powerful influences on preferences for place of death [12], but few studies have taken into account the presence or absence of a co-resident family caregiver on experiences in end of life care, and preferences for place of care. One study based on a single Australian service found that after patients have accessed specialist palliative care, people who live alone received less counselling than their peers who lived with others, but needed more social services input [13].

Many models of end-of-life care assume that older adults have friends or relatives available to provide unpaid care in the home. Current UK policy places great emphasis on ensuring that people may choose where they are cared for and subsequently die [14]. This approach is expected to reduce health care costs by increasing the proportion of home deaths [15], but the consequences of such policies for people without co-resident carers, are unknown. Ageing of the population means that providing appropriate, equitable care for older adults who live alone with cancer will be an increasingly important issue for service providers in the coming years. Without careful anticipatory planning and coordination of services, choices may be restricted for people living alone. This study was concerned with older adults coming to the end of their lives with cancer. The aim was to understand how experiences of health and social care at the end-of-life may differ with living arrangements. This paper explores and compares the experiences and preferences for place of care and place of death of people living alone, with those of people who live with others.

## Methods

Twenty-one participants were identified by their general practices; the rest by medical and nursing staff in hospice day care units. In each participating practice, records were searched to identify all registered patients aged 75 years and over with a recorded diagnosis of cancer (excluding non-melanoma skin cancers). At least one GP within each practice then restricted the list of patients to those where the GP responds ‘no’ to the question: ‘Would you be surprised if this person were to die in the next 12 months’. Further exclusions were made at the doctors’ discretion, if they felt that the patient was too unwell to be interviewed or was likely to be distressed by an approach from the researcher. People with known cognitive impairment and those living in care homes or other institutions were excluded. We were not able to include non-English speakers as no funds were available for translation.

A letter of invitation was sent from GPs to their identified patients, with study information and a postage-paid form to return to the researcher if they were interested in taking part. The research team made telephone contact with anyone who expressed interest, explained the study and arranged to meet if they wished to participate. A similar procedure for recruitment was adopted in hospice day care.

All interviews were conducted in the participant’s home or within a hospice day care unit. Interviews were semi-structured and based on an interview guide which consisted of six broad topic sections; illness experience, current circumstances and social support, quality of life, use of health and social services, end-of-life preferences and future care. Interviews were recorded and transcribed verbatim. Field notes provided additional contextual detail.

### Analysis

Interview transcripts were analysed using a framework approach [16]. Two researchers (LC and BH) familiarised themselves with the detailed content of the interview data to develop a thematic framework by reading and re-reading transcripts. This framework was developed in discussion with the research team, and was further amended after being applied to more transcripts. Definitions for the themes were developed by LC and BH and transcripts were double-coded until consensus was reached on coding. The thematic framework was applied to all data and the content of each transcript coded under the appropriate themes. Data were then inserted into a chart (spreadsheet), to provide a summary of the thematic content listed by case. The chart provided a visual summary of the dataset that enabled the research team to look across cases and themes, and identify explanations and patterns in the data. This stage allowed interpretation of the dataset as a whole and was a means of connecting the data with the original research objectives, identifying commonalities and differences between people who lived alone and those who live with others.

## Results

Thirty-two older adults took part in interviews for this study: all were living with cancer, judged to be in their last year of life and aged 70 to 95 years. The twenty interviewees who lived alone had done so for less than one to more than fifty years; a majority were widowed. The characteristics of participants are shown in Table 1. The findings draw on all of the interviews, using comparison between the participants who lived alone and others to understand the differences in experiences.

**Table 1:** Participant characteristics

	**Living alone (n=20)**	**Living with others (n=12)**
**Age** in years (median, range)	82.5 (74–95)	77.5 (70–87)
**Sex**		
*Males*	10	8
*Females*	10	2
**Education:**		
Mean number of years (SD)	10.4 (1.90)	9.17 (2.12)
**Housing**		
*Owner occupied*	13	11
*Rented from local authority*	6	1
*Rented privately*	1	0
**Main cancer diagnosis -**		
Breast	3	2
Lung	4	2
Colorectal	2	2
Prostate	3	2
Oesophagus	2	0
Other	6	4

Five main themes emerged; how patients felt disadvantaged by living alone, continuity with health professionals, informal appraisal of the care received, place of care and future planning. Direct quotations from participants have been selected to represent a significant body of data, or to illustrate views and perceptions contrary to the majority.

### Living alone with cancer at older ages: themes from the qualitative analysis

Disadvantaged by living alone

Practical – appointments, telephone systems, travel to hospital

Emotional – lack of advocate, emotional support

Relational continuity in primary care

Personal relationship

Efficient consultation

Informal quality appraisal

Place of care

Continuity

Meeting expectations

Talk to the person

Holistic rather than specialty focused

Fear of admission to care home

Inability to cope heralds death

Home is preferred

Comfort and symptom control most important

Future plans

Priority to reduce burden on family after death

No advance care planning

Uncertainty, denial

### Disadvantaged by living alone

In this study, many people who lived alone felt that their living circumstances put them at a disadvantage in their interactions with health professionals. Whilst there were few objective reasons offered as to why living alone may confer a particular disadvantage, the perception was widely shared. The drawbacks to living alone were discussed most often in relation to practical issues, such as arranging to see a GP, or travelling to hospital. The less tangible aspects of accessing care – the absence of someone to provide emotional support and advocacy, or the perceived injustice of longer lengths of stay, for example –elicited stronger feelings, but were mentioned less often than practical concerns. Often, dismay was expressed at being alone, but the consequences for each individual, not clearly articulated. For example, this 87 year old participant expressed understated concern at his own lack of family support when dealing with health professionals.

*“… they (doctors) must see lots of people on their own, there is quite a lot of people living on their own you know. Of course a lot have got family as well, which is a big help …. when you are literally on your own, you haven’t got anyone, that is a little bit awkward, I know.”* Male age 87 years, lives alone Code LA003

In contrast, another participant’s daughter described her clear perception of the need for more coordination in service provision.

I just think a bit more joined up thinking you know… if somebody had the realisation that if somebody lives on their own, then you have to be a bit more proactive, to make sure that things all come together as they should, rather than - oh we will just organise that service for you. And that will come out to you, because if my Dad is in hospital they can’t unless they communicate with us…..

Daughter of participant Code LA025, female age 80 years, lives alone.

Certain routine aspects of health services organisation were perceived by older people living alone to present barriers to solitary dwelling elders. Interviewees described how they looked for ways around obstacles. Going in person to make an appointment, for example, was felt to be a means of securing a prompt consultation. When doctors visited at home, the greater individual attention was a source of satisfaction. One interviewee reported asking for a visit even when she was able to attend the surgery, in order to have a longer consultation.

Well you can’t get through, I keep ringing the same, mind you I don’t go often, I really don’t, I can’t get through, I ring up and its engaged, will you hold and you know the same old story and I have been waiting 20 minutes and nothing has happened. And the voice keeps coming on, and music, we are attending to you, so I just slam the phone down and that has happened several times, that is why I sent for the doctor to come and see me because I wasn’t getting anywhere fast ringing up to go and see him. Mind you the surgery is not far from me, it’s down the road on the left, and you see, through him coming out, I got all that attention.”

Female, age 80 years, lives alone Code LA011

There were few data presented to support the existence of specific barriers to services for people living alone, yet those interviewees who did live alone, spoke of the difficulties they experienced. In their talk of the difficulties in accessing services, participants may be revealing their emotional responses to being alone and unsupported. Seeking greater attention from individual doctors and finding difficulty in articulating the consequences of solitary living may all support the possibility that it was a need for emotional rather than practical support that was being expressed.

The perception that doctors were reluctant to make house calls was widely shared, but there were exceptions. This 90 year old was appreciative of a responsive service from her GP but also perceived enhanced surveillance to be accompanied by the threat of loss of control over her life. Independence was prized by all, but of particular import to those who live alone.

“I can always get in touch with the surgery because if you can’t come up and visit, if I am not in a position to go down, and he will talk to me over the phone and advise, you know. Or I am only, he is only down the road, or he will pop up in his car, and check up on me but that is as far as it goes. I don’t trouble anybody, if I can get on with my life. You see you have got to be very careful when you get elderly, the minute you start to fade either physically, mentally or whatever, you know, you find that your house isn’t your own anymore, there is always somebody knocking at your door, to take control of your life. I am saying this in a kind way we are that sort of nation now, nobody wants to be held responsible for anything that happens to the elderly and I think it’s gone a bit overboard. You know I like being independent. I love, I am not saying I can go for years being like I am, independent but I am enjoying being independent, I think, they appreciate you at the surgery if you are independent.”

Female, age 90 years, lives alone Code LA019

Awareness of their advanced years was a strong theme running throughout the interviews, and age rather than living circumstances, was put forward as a marker of greater need for care and attention.

### Relational continuity in primary care and appraising the care received

Relational continuity was important to a majority of the interviewees, but particularly those who lived alone. In most cases, this was mentioned in the context of how difficult it was to see the same doctor in primary care. Two issues were apparent, the absence of a personal relationship that they might have had with a GP and how the lack of prior knowledge wasted time for both doctor and patient, as they repeated information. There was clearly an expectation of continuity in general practice, which meant that its absence was keenly felt. This did not appear to be the case for hospital attendances, where continuity of care was not anticipated.

“Well it’s getting confidence in your GP, some you get it more confidence than others, the present gentleman…….we have a bit of fun together, tell a few jokes and we are on the same wavelength and he does anything he can for me. But there is no certainty that he will be there the next time I go, because they are forever changing the doctors. You get confidence in one doctor, they know your little shortcomings and that sort of thing, and then you go next time and you have got to train the new one.”

Male age 83 years, lives with wife, Code LA007

Even without continuity, the knowledge that health professionals were accessible, was reassuring for many of the older adults who lived on their own. Described by a 75 year old female as *“Security that I am in touch with somebody who knows what they are doing, because I have no idea.” (Code LA015)*, the reassurance lies in the combination of expertise and availability. Indeed, this confidence and trust in doctors and nurses to provide services for all, was assumed to be implicit in NHS care. Older people at home were grateful for care workers or health professionals who take the time to speak with them as people, as well as recipients of an intervention or service. For some, continuity provides reassurance, but for others, it was the doctor’s knowledge or willingness to refer promptly to hospital that were valued.

“Previous to this, seeing this doctor, there was a doctor who started this practice on his own so I was always seeing him, and he was wonderful, you know, before now they can’t put their hand on you at all can they, you know they have got to be careful doctors now, but he would put his arm on me, hand on my shoulder and on my hand to reassure me, I knew when I was down I could go and see him and I would feel better. When I walked out of that surgery I felt better because he was really lovely with me, but then he sold the practice to a big practice in [town], so it’s merged, so we have got all different doctors now and now he has gone because he specialised in various things and he has gone to now to stay with the things he likes to do really rather than general practice, so he has gone, but I felt better when he was there, in a way. I knew it would be him all the time and how he treated me.”

Female age 76 years, lives alone Code LA014

Such insights and perceptions are not unique to people who live alone, but the importance of the personal aspects of care provided was emphasised more readily by solitary dwelling participants.

“I have got the numbers if I want to ring them, if I have any upset or I am worried about anything, just got to ring them up and they have both left me cards, so I am in touch if I need to be. ….. If I have got any queries I have only got to pick the phone up.”

Male age 79 years, lives alone Code LA009

“They all seem very caring. Very helpful, they keep in contact quite a lot by phone you know, if you wanted them to come round they would come round and discuss things with you, that is if it was necessary, but I have never felt the need, to do that you know”

Male age 87 years, lives alone Code LA003

### Place of care

All participants stated a preference to remain in their own homes for as long as possible. Maintaining their independence, having their own things around them and having the option to die at home, were important issues. Boundaries to family care were acknowledged, with some aspects of personal care judged not to be appropriate for a male relative to provide. Moving to live with children or other family members was generally felt to be a last resort and ensuring that they neither burdened nor interfered with their children’s lives, was an important principle that was guiding choices.

Well that would be the last resort. That’s all I can say. I have accepted this and I don’t mind living as I am now at the moment. And, when if I have to go and live with anybody it would be my niece and I wouldn’t like, you know she is a good person and she works hard, and I wouldn’t like to be a sort of a burden on her you know, so I will stay as I am as long as I can,

Because once I have to walk out of here that’s it, this is my home.

Male, age 95 years, lives alone Code LA008

I wouldn’t like to go and live with family, I love them dearly but I wouldn’t like to think I was going to spoil their lives and while I can live on my own and manage that is what I intend to do. I would rather be living here on my own than having to go and live with my daughters or go into a nursing home. And as long as I can manage here that is what I am hoping I can do.

Female, age 97 years, lives alone Code LA022

The prospect of moving into a care home was not a positive choice for any of the participants, but it was a real source of anxiety for some people who lived alone. Underlying this was a fear shared by many who lived alone, that being unable to cope would be a sign of the end-of-life. This interviewee (below) articulated concerns that were common to many, the costs of residential and nursing care, and a perception that they may not be nice places to spend one’s final days:

“I often wonder it would be like going into one of these nursing homes or whatever, I don’t think I would fancy it very much you know………. This does worry you sometimes thinking about it you know, I mean I remember going once to see a friend of mine, and it was a place by the hospital. While we were talking she was having her dinner, there was a little mouse on top of the radiator, and I thought oh …..…. You are worried about sitting at the dinner table and mixing with people who you wouldn’t say are your class, but really crude, which could happen. They are things that worry you, you know. Having to spend your life with the type of rough people.

I think when you get older, through having plenty of money, you can choose, pick and choose, what kind of place you want to go and stay in. I mean, my age now, very little savings, no way could I afford to go into a nursing home of choice, say somewhere nice, in a nice area. These things which do niggle at you now and again, they niggle at you”

Male age 87 years, lives alone Code LA003

The participants who lived with a husband or wife, expected to be able to look after each other until it became too much work for the partner, when they would be obliged to consider other options.

“We have never really sat down and said this is what we want to happen, we have never sat down and said that, but we have, I mean we both would want the best, and if we couldn’t give the best and somebody else could, well that would be how it would have to be. I don’t think we would ever reject anything, we don’t rule anything out, our basic thing is we want to stay here, but if it came to it we would have to weigh up which is the best for the other person, and if it was better for the other person to go say into a hospital or a hospice then that is what it would have to be. Because it would be the best for them, not the best for whoever is left. It would have to be that. I do tend to push it to the back of my mind but, if it came, push to shove, we do have Masonic Homes and that is where quite a lot of elderly Freemasons go, to finish their days you know, and ladies as well, it’s for ladies as well.”

Male age 83 years, lives with wife, Code LA007

### Uncertainty about the future

Our participants were all judged to be in the last year of life and aware of their prognosis. Despite this, none reported having discussed plans for their future care with health professionals. The majority felt that they would have to consider making plans for their future care only when their condition deteriorated. A few acknowledged that they may not live long enough to make such arrangements. Fluctuating conditions left a third, smaller group of people uncertain of how urgent the need to make plans really was.

“I don’t know how long it’s going to go on, how bad it’s going to get, how quickly it’s going to get I mean I keep thinking well I don’t think I will be here next Christmas, and then you know things sort of clear up and I think oh well, getting on alright. And, it’s so uncertain. I mean I have been looking at televisions and I don’t know whether to buy one or not.”

Female, age 82 years, lives alone Code LA010

Participants were concerned, in general, with remaining life, and made plans for death, but not deteriorating health. People who attended day hospice expressed a preference to die in a hospice. Others had far less firm ideas about their preferred place of death, and suggested that comfort and the absence of pain were more important to them than the location.

There were a number of participants who lived with a spouse who had not made a will, had had no discussions and expressed no interest in planning for their future. This was not the case for any of those who lived alone. Most participants, regardless of living situation, had made some preparations for their deaths, with savings, insurance policies and funeral arrangements. There was some agreement that the best time to discuss things was in advance, but only a vague idea of when this should be; not too soon, when you are not feeling well, perhaps three months before the end.

These participants described a commonly shared desire to minimise the burden on their bereaved relatives, by organising their affairs in advance of their deaths.

“I thought well, I will get it sorted because you can never tell when it will happen, so I thought, right this is what we will do, so it is all done, it’s all paid for, it’s all sorted. It’s very important because I realised how helpful my daughter was in things like probate, it’s quite simple, quite simple, but the shock that I had, made it very difficult for me to cope. So, this way my whole family now knows what the score is, they don’t have to worry about it, don’t have to think about it.”

Male age 76 years, lives alone Code LA014

“I feel as if it’s my duty. I feel as if that is the least I could do, even though those lads are in good jobs and I know they are, the eldest one especially, but I didn’t want to leave a debt. I wanted to go and it would be lovely if it was paid for.”

Female, age 74 years, lives alone Code LA013

## Discussion

Many of the experiences of older adults with advanced cancer were common to all, irrespective of living arrangements. People in this study who lived alone shared concerns over where they may be cared for in the future as their condition deteriorates, balanced by a desire to remain independent and not burden their families. These feelings had generally not been acknowledged or addressed by their professional carers, and no plans made for future health care decisions. Some of the changes made to the organisation of primary care services in the UK aimed at increasing efficiency and cost containment, were perceived as barriers to accessing health care.

### Comparison with other work

Most research that has sought the views of older adults on the services they would prefer to receive when their condition deteriorates, or future admission to long term care, has not been conducted with people who were aware that death was imminent [17-19]. In contrast, our participants had a cancer diagnosis and were living with a terminal illness. They expressed a desire not to be a burden to people who live on after their death, a finding shared with studies of older adults who are housebound [19] and those living independently [17]. Making plans for funerals was also a common theme. The desire to remain at home for as long as possible has been noted in other populations, along with a concern for comfort, rather than place of death [8,20]. Despite the attention given to advance care planning in research [21,22], the impact on health care decisions is uncertain [23] and none of the participants in our study had made these formalised plans. The process of discussion and review that would foster the development of advance care plans requires a trusting relationship with a professional carer. It may be that the lack of continuity in both primary and secondary care experienced by some participants was a significant barrier to meaningful discussion. Living alone is acknowledged as a risk factor for admission to long term care [24-26]. Hence it is not surprising that the participants who lived alone expressed the most concern about this.

Our interviewees felt disadvantaged if they lived alone, but it is important to note that we have no data to suggest that they used fewer services, or lacked access to specific forms of care. Evidence for an association between living circumstances and use of services is conflicting. Older adults living alone have not been shown to be high users of primary care [27], and services for patients with lung cancer were found to relate to need rather than living circumstances [28]. Readmission to hospital is more common amongst people living alone [29], and older people who live alone have been shown to be more likely to die in hospital [30,31]. The lower proportion of home deaths[32] could be related to the absence of co-residents [6] or in some cases, reduced access to specialist palliative care [13,30].

Living alone is not necessarily synonymous with an absence of caregivers or family members, and the network of social support available from outside the household is an important determinant of need for care. Nevertheless, the presence or absence of co-resident caregivers will influence the level and type of support services required. It may also be associated with loneliness and isolation – both of which are linked with increased morbidity and premature mortality from a range of causes [33]. In this study, feeling emotionally unsupported may have been at the root of participants’ perceptions of being disadvantaged as patients if they lived alone. It is also important to acknowledge that living circumstances may change for a number of reasons and do not have the stability of other variables used to characterise individuals, such as educational achievement or even socioeconomic status.

#### Strengths and weaknesses

The study participants were aged, unwell and many lived in disadvantaged areas. Thus they represent a group whose voice is heard less often in research. All would have lived through war or times of austerity, and the stoicism that was apparent in many of the accounts is likely to reflect their life experiences. But whether these attitudes would be shared by less aged adults living with cancer, requires further study. Older men living alone were over represented in our study. Half of our participants who lived alone were men, whereas the equivalent figure in the UK population is around 30%. Gaining insights into the experiences of this group is a strength of our study, as older men living alone may be less likely to seek help for health related problems and have fewer social ties and less emotional support than their female peers. It was important that participating GPs were able to use their clinical judgement to exclude potential interviewees, but we cannot know if there was any variation in the way inclusion criteria were applied. Research governance protocols leave us with no data on the characteristics of non-responders, or the populations from which our sample was selected. This is a limitation shared by all similar studies.

## Conclusions

Our findings appear to reinforce previous calls for more open discussion and debate about death [34]. Evidence that patients benefit from or desire discussion of death is limited, but in the UK, structured assessments such as the Gold Standards Framework already require health professionals to identify patients who are thought to be in the last year of their lives, assess needs and make plans [35]. We did not ask our interviewees directly about the GSF, but there was little talk of the outcomes of meaningful discussions and planned support. With older people who have no co-resident carer, such discussion is likely to be an essential part of supporting the widely shared determination to remain independent. Addressing the particular concerns and fears of people living on their own – admission to a care home, for example – may improve patients’ experiences and could complement other work to enhance clinical outcomes. It may also provide an opportunity to identify any perceptions of isolation or feelings of loneliness that influence quality of life. Seeing the same doctor or nurse over weeks, months or years allows relationships and trust to develop, and these are an essential basis for sensitive discussions. Finding ways to provide continuity in the rapidly changing environment of primary care, will be a major challenge. But it may prove to be crucial to improving care for older people with cancer who live alone.

## Ethical approval

Ethical approval was granted by Liverpool Central Research Ethics Committee (09/H1005/42).

## Competing interests

The authors declare that they have no competing interests

## Authors’ contributions

*BH* developed the idea for the study with *GG, SP, AA, JS and JAH. LC* conducted the interviews and analysed the data with *BH. BH* wrote the first draft of the paper. All authors have contributed to and approved the final manuscript.

## Pre-publication history

The pre-publication history for this paper can be accessed here:

http://www.biomedcentral.com/1471-2296/14/22/prepub
